# A Novel Sesquiterpene Lactone Xanthatin-13-(pyrrolidine-2-carboxylic acid) Isolated from Burdock Leaf Up-Regulates Cells’ Oxidative Stress Defense Pathway

**DOI:** 10.3390/antiox10101617

**Published:** 2021-10-14

**Authors:** Yanis A. Idres, Didier Tousch, Guillaume Cazals, Aurélien Lebrun, Sarah Naceri, Luc P. R. Bidel, Patrick Poucheret

**Affiliations:** 1 UMR 95 Qualisud, University Montpellier, CIRAD, SupAgro Montpellier, 15 Avenue Charles Flahault, BP 14491, CEDEX 5, 34093 Montpellier, France; patrick.poucheret@umontpellier.fr; 2 Laboratoire de Mesure Physique, Université de Montpellier, Place Eugène Bataillon, CEDEX 5, 34093 Montpellier, France; guillaume.cazals@umontpellier.fr (G.C.); aurelien.lebrun@umontpellier.fr (A.L.); 3 Laboratoire de Biologie Fonctionnelle et Adaptative, Université de Paris, CNRS UMR 8251, 35 rue Héléne Brion, 75013 Paris, France; sarah.naceri@etu.u-paris.fr; 4 INRA, UMR AGAP, CIRAD, SupAgro, 2 Place Pierre Viala, 34060 Montpellier, France; luc.bidel@inrae.fr

**Keywords:** burdock leaf, sesquiterpene lactones, xanthonolide, oxidative stress, glucose-6-phosphodeshydrogenase, molecular docking

## Abstract

The aim of our study was to identify novel molecules able to induce an adaptative response against oxidative stress during the first stages of metabolic syndrome. A cellular survival in vitro test against H_2_O_2_-based test was applied after pretreatment with various natural bitter Asteraceae extracts. This screening revealed potent protection from burdock leaf extract. Using chromatography and LC-MS—RMN, we then isolated and identified an original sesquiterpene lactone bioactive molecule: the Xanthatin-13-(pyrrolidine-2-carboxylic acid) (XPc). A real-time RT-qPCR experiment was carried out on three essential genes involved in oxidative stress protection: GPx, SOD, and G6PD. In presence of XPc, an over-expression of the G6PD gene was recorded, whereas no modification of the two others genes could be observed. A biochemical docking approach demonstrated that XPc had a high probability to directly interact with G6PD at different positions. One of the most probable docking sites corresponds precisely to the binding site of AG1, known to stabilize the G6PD dimeric form and enhance its activity. In conclusion, this novel sesquiterpene lactone XPc might be a promising prophylactic bioactive agent against oxidative stress and inflammation in chronic diseases such as metabolic syndrome or type 2 diabetes.

## 1. Introduction

Aerobic cells generate chemical energy through carbohydrates and lipids oxidation using dioxygen (O_2_) consequently reduced into water. Incomplete reduction of O_2_ generates various reactive oxygen species (ROS) such as superoxide anion (O_2_^●−^), hydrogen peroxide (H_2_O_2_), and hydroxyl radical HO^●^ [1,2,3]. ROS are highly reactive and can induce deleterious oxidative damage to DNA, protein, and lipid peroxidation at high concentrations [4]. To maintain physiological cellular levels of ROS and oxidation-reduction (red/ox) homeostasis, cells have developed a physiological survival detoxification process involving several endogenous molecules such as glutathione, and enzymes such as superoxide dismutase (SOD) or glutathione peroxidase (GPx) [1,4].

The endocellular glutathione in its reduced form (GSH) is the first defense against ROS. In fact, GSH is mobilized in a red/ox reaction conducted by glutathione peroxidase (GPx) to generate ROS of lower reactivity and toxicity for cells, and the disufide-glutathion oxidized form (GSSG). GSH depletion must be replenished, both by a de novo synthesis [5] and by regeneration of GSH regulated by glutathione reductase with NADPH as reducing cofactor [6,7]. The latter is mainly provided by the glucose-6-phosphate dehydrogenase (G6PD), the first rate-limiting enzyme of the pentose-phosphate pathway, which is the main source of cellular NADPH [8,9]. Therefore, G6PD plays a crucial role both in ensuring protection against oxidative stress and allowing protein and lipid synthesis.

It is well known that a state of imbalance between ROS generation and ROS neutralization by antioxidant systems of cells and tissues is a key factor in the etiology of metabolic diseases and cardiovascular diseases (CVDs). This homeostasis disturbance is defined as oxidative stress. More specifically, it is involved in pathologies such as metabolic syndrome (MetS) and type 2 diabetes (T2D). In MetS, a high flow of free fatty acids and glucose is associated with the increased production of mitochondrial ROS and subsequently with increased oxidative stress [10,11]. Cellular suffering induces the secretion of pro-inflammatory factors promoting chronic inflammation, and insulin resistance in insulin sensitive cells, both contributing to the onset and development of T2D. In this context, several studies have shown, in in vitro and in vivo models of MetS, a decrease in the activity of antioxidant enzymes [10,11,12]. Increased ROS production should be considered a primary and/or a secondary cause of the CVDs [13]. Furthermore, it now recognized that a deficiency in G6PD is associated with an increased risk of T2D. Hence, recovering redox homeostasis may be a useful approach to prevent the MetS.

To found new natural compounds able to activate protective pathways against excessive oxidative stress, it seems that bitter Asteraceae plant family could be a good candidate. Indeed, these plants are known for their antioxidant potential, attributed to their significant content in phenolic compounds and more particularly to hydroxycinnamic acids and sesquiterpene lactones (SL) [14]. Often described in the literature, phenolic compounds are antioxidant, both for their hydroxyl reducing functions and for their pleiotropic actions modulating the activity of cyclooxygenase (COX), lipoxygenase (LOX), and nitric oxide synthase (NOS), known to be involved in inflammation processes [15]. Moreover, some of these phenolic compounds, such as anthocyanin, can upregulate the expression of SOD and GPx and attenuate lipid peroxidation [16].

Many anti-inflammatory mechanisms of action of some sesquiterpene lactones have been reviewed [17]. SL behave as electrophile acceptors and react according to a Michael addition reaction, with some cysteine sulfhydryl groups of protein being involved in transcriptional complexes, in particular (Keap1) and p65. Among them, several SL inhibit Nuclear Factor kappa-B (NF-κB) induction by targeting p65 subunit [18,19]. Moreover, some of them inhibit cytokine induction by targeting Tumor Necrosis Factor alpha (TNF-α), and Janus kinase (JAK)/Signal Transducer, as well as Activator of Transcription (STAT), leading to a significant anti-inflammatory effect. In contrast, sesquiterpene lactones can also activate the nuclear factor-erythroid-2-Related Factor 2 (Nrf2) through dissociation from Kelch-like ECH-associated protein 1 (Keap1) [17]. Being released, Nrf2 can enter the nucleus to engage in transcription of detoxification enzymes such as heme oxygenase-1 (HO-1), superoxide dismutase (SOD), glutathione peroxidase (GPx), and glutathione S-transferase (GST) [20].

In the present study, we selected bitter Asteraceae plants; for each of them, we prepared a LH20 hydroalcoholic extract in order to perform a cellular screening aiming at evaluating their protective effect against H_2_O_2-_generated oxidative stress. The burdock leaf extract showed the most potent protection. We then identified a potent molecule responsible for this protective effect and performed RT-qPCR of a few genes involved in oxidative response as well as a molecular docking approach to determine the biological target of this new sesquiterpene lactone molecule.

## 2. Materials and Methods

### 2.1. Materials

All plants were collected from the Mediterranean garden of Roquebrun (France) during the months May, June, and July, except burdock, which was collected in September. The Asteraceae collection comprised *Arctium lappa L.* (Burdock), *Cichorium intybus L.* (Chicory), *Cichorium endivia* (Escarole), *Artemisia dracunculus L*. (Tarragon), *Inula helenium L.* (Elecampane), *Taraxacum officinale L*. (Dandelion), *Cynara Scolymus L*. (Artichoke), and *Xerochrysum bracteatum L*. (Strawflower).

Plants were identified by botanical biologists (burdock: A Voucher specimen was deposited in the botanic garden, HBH071). The parts of plants were dried at 60 °C in a dark room.

L6 rat myocyte cells (LGC Promochem, Molsheim, France) were cultured in Dulbecco’s modified eagle medium (DMEM) with 10% fetal calf serum (FCS) and supplemented with a cocktail medium composed of 2 mM L-glutamine, 100 U·mL^−1^ penicillin, and 100 µg·mL^−1^ streptomycin (Sigma-Aldrich, Munich, Germany). The primers used for real-time RT-PCR (qPCR) were synthesized by Sigma-Aldrich (Munich, Germany).

### 2.2. Preparation of Plant Extracts

A crude extract was obtained after drying and grinding the aerial part of the plant using the method previously described by [21] with some modifications. Briefly, 50 g of the leaf powder was placed in a cellulose capsule and macerated in a volume of EtOH-Water (70:30) for 12 h. After being concentrated, the crude extract was placed on a low-pressure liquid chromatographic column with LH-20 adsorbent (Sigma-Aldrich, Munich, Germany). The elution procedure consisted of an ethanol gradient ranging from 20 to 90%. The eluate collection began around 40% of EtOH up to 60%. All the extracts collected were subjected to an H_2_O_2_ survival test.

### 2.3. Preparative UPLC-HRMS-MS to Obtain a Pure Extract of the Active Molecule

For chromatographic separation and mass spectral analysis, an Acquity H-Class UPLC system (degasser, quaternary gradient pump, autosampler, column thermostat, and diode array detector) was used coupled with a Waters Synapt G2-S LC/MS system equipped with ESI ion source (Waters, Milford, CT, USA). UPLC separations were achieved on a Kinetex EVO C18 1.7 µm (100 mm × 2.1 mm i.d.; Phenomenex, Torrance, USA). Mobile phase consisted of 0.1% (*v*/*v*) aqueous formic acid (A) and acetonitrile supplemented with 0.1% (*v*/*v*) formic acid (B). The following gradient program was applied: 0.00 min, 5% B; 10.00 min, 100% B; 10.10 min, 5% B; and 12.00 min, 5% B. The solvent flow rate was 0.5 mL·min^−1^, and column temperature was set at 25 °C. The injection volume was 1 μL.

Electrospray conditions were as follows: drying gas (N2) temperature, 450 °C; flow rate, 12 L·min^−1^; nebulizer pressure, 6 bar (N2); cone voltage, 30 V; and capillary voltage, 3000 V. Full scan mass spectra were recorded in positive or negative ion mode over an *m*/*z* range of 50–1200 MSMS.

Purification was achieved on semi-preparative HPLC-DAD Ultimate 3000 (Thermo Fischer Scientific, San Jose, CA, USA) equipped with a Kinetex C18 5 µm (250 mm × 10 mm i.d; Phenomenex). Mobile phase consisted of 0.1% (*v*/*v*) aqueous formic acid (A) and acetonitrile supplemented with 0.1% (*v*/*v*) formic acid (B) with isocratic separation under 10% B during 60 min at a flow rate of 3 mL/min.

### 2.4. NMR Analysis

NMR spectra were recorded at 298 K on a Bruker Avance III 600 MHz NMR spectrometer, using TCI Cryoprobe Prodigy^®^. Bruker Biospin Fällanden, Switzerland) Chemical shift data are given in δ ppm calibrated with residual protic solvent (e.g., MeOD4: 3.31 ppm—1/49.0 ppm—13C). 2D homonuclear 1H COSY, TOCSY, and ROESY (2–32 scans, 512 real (t1) × 2048 (t2) complex data points) and 2D heteronuclear spectra 13C g-edited HSQC and 13C g-HMBC were acquired to assign the compound (16–64 scans, 256–512 real (t1) × 2048 (t2) complex data points). Spectra were processed and visualized with Topspin 3.6.2 (Bruker Biospin Fällanden, Switzerland) on a Linux station. MeOD4 was purchased from Sigma Aldrich. 1H NMR (600 MHz, MeOD4) δ (ppm): 7.20 (d, J = 16.1 Hz, 1H, 2-H), 6.36 (dd, J = 9.1, 3.0 Hz, 1H, 5-H), 6.26 (d, J = 16.1 Hz, 1H, 3-H), 4.59 (ddd, J = 12.7, 10.3, 2.8 Hz, 1H, 8-H), 3.97 (dd, J = 9.2/6.0 Hz, 1H, 16-H), 3.87 (ddd, J = 11.5, 7.4, 3.6 Hz, 1H, 19-H), 3.53 (dd, J = 13.6/3.3 Hz, 1H, 13-H), 3.47 (dd, J = 13.6, 7.9 Hz, 1H, 13-H), 3.27 (m, 1H, 19-H), 3.14 (m, 1H, 10-H), 3.05 (ddd, J = 12.7, 7.9, 3.3 Hz, 1H, 11-H), 2.75 (ddd, J = 16.5, 9.1, 2.3 Hz, 1H, 6-H), 2.46 (m, 1H, 17-H), 2.36 (m, 1H, 6-H), 2.34 (ddd, J = 12.7, 4.1, 2.8 Hz, 1H, 9-H), 2.30 (s, 3H, 15-H), 2.19 (m, 1H, 17-H), 2.14 (m, 1H, 18-H), 1.98 (m, 1H, 18-H), 1.96 (m, 1H, 7-H), 1.75 (td, J = 12.7, 3.8 Hz, 1H, 9-H), 1.20 (d, J = 7.4 Hz, 3H, 14-H); 13C NMR (150 MHz, MeOD4) δ (ppm): 201.6, 177.9, 173.1, 150.7, 146.3, 140.8, 125.4, 83.4, 71.8, 57.1, 54.7, 48.9, 45.0, 37.0, 30.2, 29.8, 28.4, 27.6, 24.3, 18.7.

### 2.5. In Vitro Test Protection against H_2_O_2_ on L6 Cells

The procedure previously described [22,23,24] was used with some modification. Briefly, L6 cells were seeded at 3 × 10^3^ cells per well in 96-well plates in DMEM culture medium supplemented with FCS and cocktail medium. After four days, the culture medium was changed and supplemented with the different Asteraceae extracts (100 µg·mL^−1^) and controls (Vitamin C or Quercetin at 10 µg·mL^−1^). After an incubation of 12 h, the cells were washed with Krebs-Ringer bicarbonate buffer with 0.1% BSA (pH 7.4) and placed in the same buffer (200 μL per well) with or without H_2_O_2_ at 60 μM. After two hours of incubation at 37 °C, the cells were washed once with PBS and incubated for 5 min in 100 μL of trypan blue solution diluted four times in PBS. The trypan blue solution was removed, and the cell layer was visualized under a microscope for large fields at 16× zoom and 40% magnification. Images were scanned, checked, and selected to estimate cell death density per well and compared to reference wells without H_2_O_2_ using ImageJ V1.48 software according to the user guide (https://imagej.nih.gov/ij/docs/guide/user-guide.pdf revised in 2018: https://cpb-usw2.wpmucdn.com/voices.uchicago.edu/dist/c/2275/files/2020/01/ImageJ_Basics_revised_for_2018.pdf) (accessed on 5 October 2021). Briefly, filters were applied to subtract the background and images were converted to black and white where blue cell nuclei were translated in black spots without gray level. The densities of spots were calculated and data expressed as percentage of mortality.

### 2.6. Real-Time RT-PCR (qPCR)

Total RNA was purified from pre-seeded L6 cells (2 × 10^5^ cells) with and without the active molecule XPc using the Promega SV total RNA Isolation system Ref Z3100 (Promega, Madison, USA). For cDNA synthesis, 1ng of RNA was reverse-transcribed using the Promega Reverse transcription system Ref A3500 (Promega, USA). PCR amplification was then performed using specific primers for SOD, GPx, and G6PD. Endogene β-actin gene has been used for normalized the PCR. The oligonucleotide design was made with the sequence of the mRNA: direct and reverse primers 52032-TTCAACCCCAGCCATGTA-3′; 5′-GTGGTGGTGAAGCTGTAGC-3′ (accession number NM_031144.3) for β-Actin, 5′-CCAGCCTCCTACAAGCACCTCAACAG; 5′-CGACAGTTGATTGGAGCTCTGCAGG-3′ (accession number NM_017006.2) for G6PD, 5′-CACTTCGAGCAGAAGGCAAGCGC-3′; 5-CACATTGCCCAGGTCTCCAACATG-3′ (accession number BC082800.1) for SOD and 5-GTCGCGTCCCTCTGAGGCACC-3′; and 5′-GAGCGGGTGAGCCTTCTCAC-3′ (accession number NM_030826.4) for GPx. The reactions were performed in a final volume of 25 μL containing 5 μL cDNA, 400 nM primer pairs using the qPCR Master Mix kit, Ref 600828 (Agilent technologies, Santa Clara, USA). PCR conditions were as follows: the polymerase was pre-activated for 90 s at 95 °C, and amplification was performed by a 40-cycle PCR (95 °C/30 s; 60 °C/60 s; and 72 °C/60 s). Analyses were performed on the Mx3000P^®^ qPCR Thermocycler (Stratagene, San Diego, USA). Ct was been estimated by the amplification curves. Specificity of the amplification was verified on the dissociation curves. The relative quantification using β-actin gene as reference was assessed with the delta Ct method by calculating the value 2^−ΔΔCt^ method described by [25]. This relative quantification method allows the evaluation of a PCR of a target transcript in treated sample in comparison to that in an untreated control throught normalization with the β-actin transcript as reference. The calculation used was as follow: −ΔΔ Ct = (Ct.target − Ct.β-actin)_XPc treated_ − (Ct.target − Ct.β-actin)_untreated_. PCR products were also submitted to an electrophoresis in agarose gel 2% in TAE to confirm the length of the only DNA amplified (data not shown). Previously, amplification curves in function of cDNA concentrations permitted one to ensure identical PCR efficacy for the target genes studied.

### 2.7. Molecular Docking Simulation

AutoDock is a molecular docking program that has been developed to predict the interactions that may exist between small molecules and proteins. The goal is to find the global minimum of the interaction energy between a small molecule (the ligand) and the targeted protein (the receptor). The program will force the system to explore the conformational space by varying all possible degrees of freedom of the ligand (dihedral angles, rotations, translations, etc.) while keeping the protein fixed. For each conformational variation, the resulting interaction energy is evaluated [26]. In the present study, we used the docking program Smina a fork of AutoDock Vina (Version 1.5.6), especially optimized to support high-throughput scoring and user-specified custom scoring functions [27].

#### 2.7.1. Preparation of the G6PD (2BHL) Protein Receptor

We downloaded the protein G6PD from PDB (Protein Data Bank) [28] in pdb format (pdb code: 2BHL). The water and (6-*O*-phosphono-beta-d-glucopyranose and glycerol) molecules that were found in the crystallography files were removed.

We need to obtain a file compatible with the autodock software; it must contain:

- Polar hydrogen atoms, as they are involved in hydrogen bonding.
- Partial charges on the atoms to simulate electrostatic interactions.

#### 2.7.2. Preparation of the Ligand Input File

The ligand (XPc) was converted with the openbabel software [29], in PH 7.4 from mol file to pdbqt format. The latter is similar to pdb but also contains information on the torsions that must be active and the assigned load for each atom. Open Babel allows one to generate a 3D structure from connectivity information. The gen3D option allows one to generate several conformers for the same molecule. The OBGastChrg is responsible for the assignment of partial charges to a molecule according to the Gasteiger charge model (sigma).

#### 2.7.3. Docking Simulation

To dock, Autodock Vina walks through the space randomly. Each position of the ligand in the receptor leads to a possible complex. Autodock Vina calculates an approximation of the free enthalpy of formation of this complex thanks to a scoring function. The best score corresponds to the answer given by the software. In our case, the ligand has covered the whole surface of the protein.

### 2.8. Statistical Analysis

Statistical analyses were performed using analysis of variance. In vitro data are expressed as mean ± standard deviation (SD). The difference was considered significant at *p* < 0.05 (*), *p* < 0.01 (**), or *p* < 0.001 (***) using XLSTAT software.

## 3. Results

### 3.1. In Vitro Test Protection of L6 Cells against H_2_O_2_ to Screen Eight Asteraceae Extracts

In order to test the protective effect against H_2_O_2_-induced artificial oxidative stress of the different Asteraceae LH-20 extracts, L6 muscular cells were pretreated for 12 h with and without different Asteraceae or the positive control (Vitamin C).

**Figure 1:**
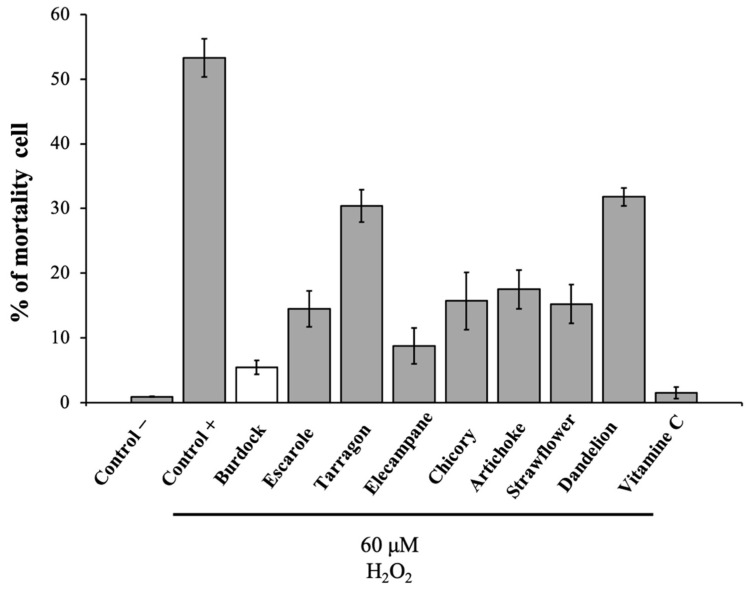
Protective effect of eight bitter Asteraceae fraction (100 µg·mL^−1^) against artificial oxidative stress induced by H_2_O_2_ on L6 myocytes compared to Vitamin C (10 µg·mL^−1^) as positive control. Treatment was applied for 12 h before the H_2_O_2_ application at 60 µM (excepted control – ).

The addition of a lethal concentration of H_2_O_2_ (60 µM) for 1 h led to more than 50% dead cells (53.3% ± 5.89). Pretreatment with the various LH-20 fractions of Asteraceae significantly reduced H_2_O_2_-induced cells death. The first screening identified burdock leaf extract as having the higher protective effect, over than 80%, with only 5.5% ± 2.2 of mortality (Figure 1) close to Vitamin C used as reference.

### 3.2. Isolation and Identification of the Protective Compound

#### 3.2.1. Isolation of Active Compound

**Figure 2:**
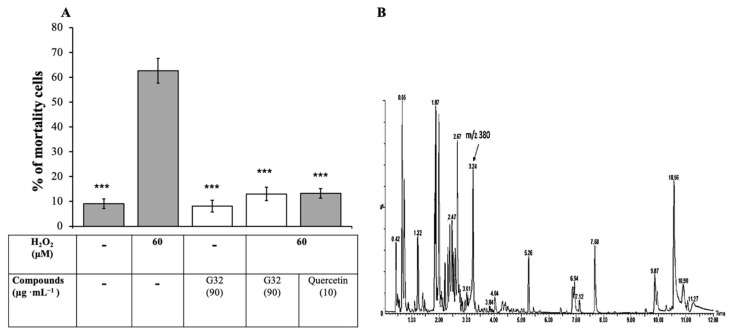
Cellular protective effect and LC-MS of the G32 fraction: (**A**) Protective effect of G32 fraction against artificial oxidative stress induced by H_2_O_2_ on L6 muscle cells. Comparison was caried out with Quercetin as positive control. Values are the means (±SD) (***) *p* < 0.001); (**B**) total ion current (TIC) chromatogram of fraction G32 in positive electrospray mode.

The LH-20 burdock leaf extract was further separated on preparative LC-MS. The fractionation resulted in an active G32 extract (Figure 2A). The UPLC-HRMS/MS analysis of active fraction G32 showed a peak of interest at *m*/*z* = 380 [M + H]^+^ (Figure 2B). HRMS analysis suggested that the formula for this molecule is C_20_H_29_NO_6_.

#### 3.2.2. Structural Elucidation

**Figure 3:**
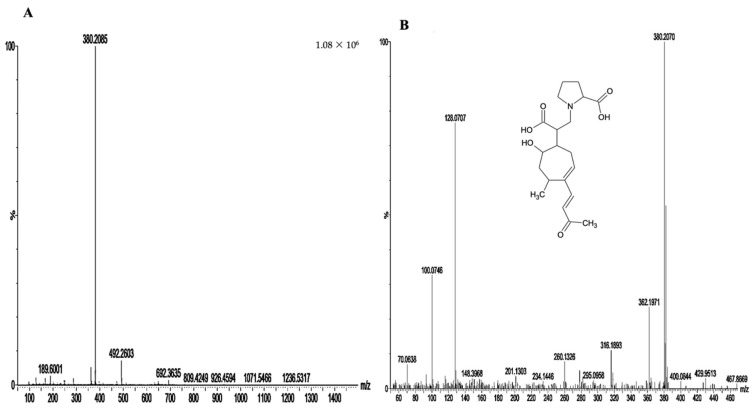
Chemical analysis of the G32 fraction and isolation of the peak of *m*/*z* 380 [M + H]^+^ molecule: (**A**) MS spectra at 3.2 min in G32 fraction; (**B**) MSMS spectra of ion *m*/*z* = 380 [M + H]^+^.

Upon UPLC-HRMS/MS and HRMS analysis cited above and identification of the molecule formula as being C_20_H_29_O_6_, fragmentation was performed. The parent ion was fragmented into *m*/*z* = 362 [M-H_2_O + H]^+^, *m*/*z* = 316 [M-H_2_O-HCOOH + H]^+^ (Figure 3). Therefore, the fragmentation pattern induced, in addition to a loss of water (*m*/*z* 362), a subsequent loss of HCOOH (316).

In the small mass parts of the spectrum, we observed a couple of ions (*m*/*z* 128 and *m*/*z* 100) that suggested a proline fragmentation pattern. We isolated this compound using semi-preparative HPLC-DAD for structural elucidation by NMR. Proline C-terminal group was determined easily with 2D-TOCSY and 13C HSQC experiments. Identification of Xanthatin part was realized thanks to 2D-COSY, 2D TOCSY, 13C HMBC, and previous literature report [30]. 13C HMBC analysis also showed correlations between C-16, C-19, and H-13, which validated junction of Xanthatin on N-proline site. Stereochemistry of chiral sites was determined by 2D ROESY. Methyl group (H-14) correlates with methyn H-8 and the latter also with methyn H-11.

**Figure 4:**
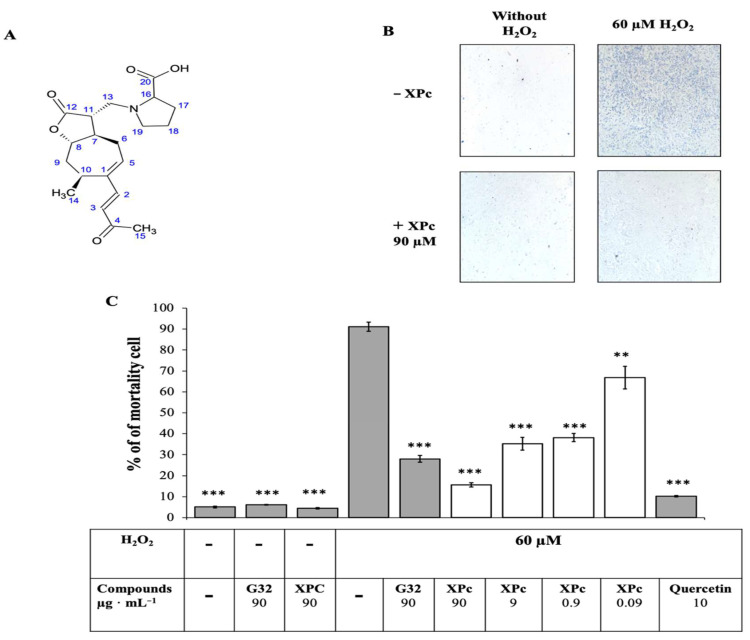
Chemical structure and H_2_O_2_ protection effect of XPc: (**A**) structure of XPc (Xanthalin-13-(pyrrolidine-2-carboxylic acid)); (**B**) images of L6 cells after trypan blue dye treatment with and without induction of H_2_O_2_ oxidative stress and with or without 12 h pretreatment with XPC; and (**C**) protective effect of XPc at different concentrations against artificial oxidative stress induced by H_2_O_2_ on myocytes comparison with G32 extract. Quercetin used as positive control. Values are the means (±SD) (*p* < 0.01 (**), (***) *p* < 0.001).

Finally, the pure molecule was identified as a Xanthatin-13-(pyrrolidine-2-carboxilic acid) (XPc). It is a derivative of Xanthathin (first isolated from *Xanthium pennsylvanicum*) the most common Xantholide, belonging to subclasses of sesquiterpene lactones (SL). It was characterized by a non-cyclic carbon chain and a seven-membered ring, in addition to the lactone ring common to all SL. XPc cellular protective effect was tested at different concentrations against artificial H_2_O_2_-induced oxidative stress. Results confirmed data from the first global screening and showed concentration-dependent activity (Figure 4). Furthermore, no toxicity was observed with XPc molecule.

### 3.3. Research of XPc Biological Targets

#### 3.3.1. RT qPCR

The biological effect of XPc as a protective compound against oxidative stress has led us to study its potential effect on the expression of genes involved in antioxidant defense. For this purpose, we have quantified the expression of SOD, GPx, and G6PD genes using real-time RT-qPCR on L6 muscle cells pre-treated or not by XPc. The results showed (Table 1) an approximately two-fold overexpression of G6PD, while no overexpression was observed for SOD and GPx. The absence of H_2_O_2_ in the experimental design excludes the possibility of an up-regulation of gene by H_2_O_2_. We have a specific stimulation of the gene expression by XPc.

#### 3.3.2. Molecular Docking

The specific overexpression of the G6PD gene led us to deduce an independent activation pathway of the transcription factor Nrf2, which is known to activate all three genes. We expected that the high anti-oxidant effect observed would not be related alone to the moderate increase of G6PD mRNA. Previous works have shown that an over-expression of the gene is concomitant to an increase of the G6PD activity [31]. It was therefore conceivable that XPc could act as an activator of G6PD with an increase of the gene transcription as a consequence. For this purpose, we evaluated the probability of a ligand–protein interaction using a molecular docking technique with Autodock Vina program.

**Figure 5:**
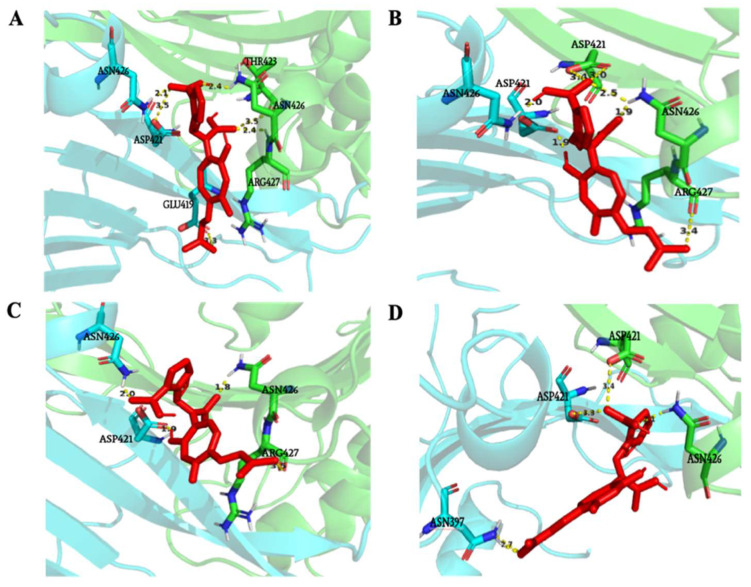
Analysis of the four possible interactions between ligand (in red) and the interface domains of the two protein monomers (blue and green): (**A**–**D**) represents the four putative positions between XPc and G6PD at the dimeric interface.

The results of the protein–ligand interaction (XPc) showed 19 poses with propitious interaction energies ranging from −8 to −5 kcal·mole^−1^. Among them, four poses with favorable energy interaction at the interface domains of the two G6PD monomers simultaneously were identified (Figure 5, Table 2). Several small molecules with similar interactions and binding energies have been previously described as increasing the activity of the enzyme by stabilizing the active hetero-dimeric G6PD [32,33]. Moreover, G6PD was described to be active in an oligomeric state [34]. As a result, our protein–ligand interaction (XPc) suggests that XPc may enhance G6PD enzymatic activity by promoting oligomerization and enzyme stability.

## 4. Discussion

The first stages of a metabolic syndrome onset are critical, as in many metabolic disorders. They may represent a target of choice for a preventive intervention to avoid co-morbidities such as type 2 diabetes. One of the central biological disruptions in the MetS etiology is oxidative stress related to an overproduction of ROS in adipose tissues [35,36,37]. Hence, a preventive strategy of interest could be the use of natural anti oxidative compound included in the daily diet. The present study aimed at searching for an efficient natural bioactive molecule bearing the potential to protect cells against oxidative stress. Bitter Asteraceae plants were selected for their high hydroxycinnamic acid derivatives and sesquiterpene lactones contents.

The first step of the work allowed to screen some hydroalcoholic bitter Asteraceae extracts through in vitro testing, which consisted of evaluating the cellular protection potential of each extract against an artificial oxidative stress inducted by H_2_O_2_. Burdock leaf showed the most potent protective effect. The combination of both sequential extraction and biological testing has led us to the purification of a new sesquiterpene lactone Xanthatin-13-(pyrrolidine-2-carboxilic acid) (XPc) demonstrating a high level of protection against H_2_O_2_ induced oxidative stress. Xanthatin was first isolated from *Xanthium pennsylvanicum* [38] and has been described as having anti-inflammatory and anti-cancer properties [30,39,40]

Xanthatin biological activity has been attributed to the presence of an exocyclic double bond, which acts as an alkylating agent and allows for the binding of many nucleophilic compounds, particularly cysteine residues of many active proteins [17,41,42]. To our knowledge, XPc is the first natural amino derivative of Xanthathin isolated from plants. However, several amino derivatives of Xanthatin have been synthesized, as mentioned in the literature [30], and showed higher water solubility and higher fungicidal activity than Xanthatin. In the literature, Xanthatin was described to promote apoptosis via inhibiting thioredoxin reductase [38].

The second part of our work investigated the cellular target involved in the protective effect of XPc against oxidative stress. A transcriptomic approach by real-time RT-qPCR in three major genes of cell defense against ROS—GPx, SOD, and G6PD—was conducted. The choice of the genes studied in the present investigation was suggested by the literature, which described various regulation pathways with differential expression patterns in response to cellular oxidative stress [43,44,45,46]. The results of this quantification showed a specific and moderate two-fold overexpression of G6PD alone. The observed G6PD overexpression observed is in conformity with previous reports [31], that established the correlation between the gene overexpression and the G6PD protein level in human hepatoma cells under H_2_O_2_ exposure. The G6PD regulation has been largely described in the literature. It is recognized today that two regulations take part in the modulation of the G6PD activity in response to an oxidative stress. Two mechanisms are proposed: firstly a transcriptional regulation increasing the G6PD level and secondly part a post-traductional regulation allowing to convert inactive to active G6PD [31,47].

The specific overexpression of the G6PD gene led us to deduce an independent activation pathway of the transcription factor Nrf2 known to activate all three genes [43,48] and appears original compared with that described in the literature. Actually, some different sesquiterpene-lactones from *Calea urticifolia*, parthenolide from *Tanacetum parthenium,* and the vernomelitensin and onopordopicrin from *Onopordum illyricum L*. have been described as Nrf2 activators and are able to elicit increased resistance to oxidative stress [20,49]. Nrf2 transcription factor has a very large spectra of action with at least a hundred target genes known to be involved in both ROS cytoprotective effect; it also regulates the glucose metabolism by mediating transcription of components of the pentose phosphate pathway [45,50]. A recent work showed the presence of onopordopicrin in the burdock leaf [23]. The presence of these two sesquiterpene lactones in burdock leaf allowed one to consider the plant as a powerful anti-oxidative stress supplement. An interesting feature of XPc effect should be emphasized: it seems to have a specific target, unlike onopordopicrin. In addition, the overexpression of Nfr2 gene target must lead to harmful effects. Indeed, it was reported that Nrf2 overexpression may increase the risk of cell proliferation [45,51,52].

The moderate stimulation of G6PD overexpression suggests a G6PD post-traductional mechanism. Considering the literature, increased G6PD activity induced by oxidative stress is essentially a genetic regulation [53] with a moderate post-traductional effect [31]. However, other treatments such as chemicals with, for example DENA carcinogen induce a strong G6PD activation at a post-traductional level in pre-(neoplastic) lesions in rat liver [54]. To test this hypothesis, a molecular docking approach allowed us to identify four favorable energy poses interacting with the dimer interface of both monomers. Similar binding positions were previously described as activating the enzyme activity by some small molecules such as AG1 molecule [32,33]. Moreover, the amino acid residues involved are exactly similar to expected ASN-397, which was specific to XPc interaction. G6PD is a hetero-enzyme that is active only in the oligomeric form; therefore, promoting stability by oligomerization can increase the activity of the enzyme [34]. In consequence, our results suggested that XPc is able to stimulate the G6PD enzymatic activity by a direct molecular interaction on the site previously described for stabilizing G6PD dimer in its enzymatically active configuration. Previous work [55] has shown that AG1 has a protective effect on erythrocyte cells when exposed to oxidative stress induced by chloroquine or diamide. In addition, it should be noted that transgenic mice overexpressing G6PD genes are protected from ageing through increased protection against oxidative damage [56,57]

It is now recognized that an increase in G6PD activity leads to GSH regeneration by reduction of oxidized glutathione (GSSG) [53]. However, another pathway promoting an increase of GSH level could be a de novo synthesis [5]. The glutamine cysteine ligase (GCL) is the first enzyme of this synthesis pathway. Like G6PD, this enzyme is an heteroenzyme structured by two self-auto-assembled subunits: a catalytic subunit (GCLC) and a modulator subunit (GCLM) [58]. Although GCLC can provide catalytic activity of its own, it has been shown that the holoenzyme increased catalytic activity [59,60].

It can therefore be assumed, as for G6PD, that the XPc compound could promote dimerization and lead to a better stability of the enzyme.

On the other hand, SLs are known to induce depletion of intracellular GSH via exocyclic double bonds, which act as alkylating function, allowing binding to cysteine-sites and leading to cytotoxicity [42,61]. However, in our cellular experiments, we have never observed any toxicity associated with XPc, and we can thereby assume that XPc does not cause GSH depletion. We support this last proposition based on the specific properties of XPc related to its molecular structure. Indeed, the presence of pyrrolidine-2-carboxilic acid on carbon 13 of the lactone group may block the alkylating function and prevent molecular interactions.

## 5. Conclusions

In conclusion, our study led to the identification of an original sesquiterpene lactone from burdock leaf, the Xanthatin-13-(pyrrolidine-2-carboxilic acid) (XPc), demonstrating a protective effect against artificially-induced H_2_O_2_ oxidative stress on L6 muscle cells. Further studies are needed to explore putative additional targets of XPc, including the enzymes involved in the de novo glutathione synthesis, and to evaluate the GSH level in cells pretreated by XPc. Moreover, structural activity studies are needed to assess the impact of proline on the protective effect of XPc and to demonstrate the direct impact of XPc on G6PD enzyme and its activity by in vitro experiment. Furthermore, it will be of interesting to assess XPc therapeutic potential in G6PD-deficient patients as proposed for AG1 and its derivatives [32,33,55] as well as in the predisposed metabolic syndrome patients. 

## Figures and Tables

**Table 1:** Quantification result of target genes by real-time PCR by the delta Ct method.

	SOD	G6PD	GPx
ΔΔCt ± SD	−0.14 ± 0.11	−0.75 ± 0.12	0.63 ± 0.21
2^−ΔΔCt^ ± SD	1.10 ± 0.09	1.69 ± 0.15	0.66 ± 0.14

**Table 2:** Results of the G6PD-ligand molecular docking scores Kcal·mole^−1^ at the interface domains of dimers and amino residues involved.

Pose	Affinity (Kcal·mole^−1^)	Amino Acids of Monomer 1	Amino Acids of Monomer 2
Pose A	−6.4	ASN-426	THR-423
		ASP-421	ASN-426
		GLU-419	ARG-427
Pose B	−5.8	ASN-426	ASP-421
		ASP-421	ASN-426
			ARG-427
Pose C	−5.6	ASN-426	ASN-426
		ASP-421	ARG-427
Pose D	−5.1	ASP-421	ASP-421
		ASN-397	ASN-426

## Data Availability

All data are included on this paper.
